# Prescription patterns in DMEK: European survey

**DOI:** 10.1097/j.jcrs.0000000000001726

**Published:** 2025-10-23

**Authors:** Yexin Ye, Fabio de Rooij, Nicolas Alejandre, Frank J.H.M. van den Biggelaar, Tristan Bourcier, Béatrice Cochener-Lamard, Francisco C. Figueiredo, David J. Galarreta, Jesper Ø. Hjortdal, Gary L.A. Jones, Naomi Nathan, Rudy M.M.A. Nuijts, Vito Romano, Andreia M. Rosa, Berthold Seitz, Marie-José Tassignon, Katrin Wacker, Mor M. Dickman

**Affiliations:** From the University Eye Clinic, Maastricht University Medical Center+, Maastricht, the Netherlands (Ye, de Rooij, van den Biggelaar, Nuijts, Dickman); Ophthalmology Department, Fundación Jiménez Díaz University Hospital, Madrid, Spain (Alejandre); Department of Ophthalmology, New Civil Hospital, Strasbourg University Hospital, FMTS, University of Strasbourg, Strasbourg, France (Bourcier); Ophthalmology Department, CHU Morvan, University Hospital of Brest, and University of Bretagne Occidentale (UBO), Brest, France (Cochener-Lamard); Department of Ophthalmology, Royal Victoria Infirmary, Newcastle upon Tyne Hospitals NHS, Foundation Trust, Newcastle Upon Tyne, United Kingdom (Figueiredo); Bioscience Institute, Faculty of Medical Sciences, Newcastle University, Newcastle Upon Tyne, United Kingdom (Figueiredo); Department of Cornea and Refractive Surgery, Instituto Oftalmológico Recoletas, Valladolid, Spain (Galarreta); Department of Ophthalmology, Aarhus University Hospital, Aarhus, Denmark (Hjortdal); The Veneto Eye Bank Foundation, Venice, Italy (Jones); Dutch Transplant Foundation (NTS), Leiden, the Netherlands (Nathan); Department of Medical and Surgical Specialties, Radiological Sciences, and Public Health, Ophthalmology Clinic, University of Brescia, Brescia, Italy (Romano); Department of Ophthalmology, Centro Hospitalar e Universitário de Coimbra (CHUC) and Faculty of Medicine, University of Coimbra, Coimbra, Portugal (Rosa); Department of Ophthalmology, Saarland University Medical Centre, UKS, Homburg, Germany (Seitz); Department of Ophthalmology, Antwerp University Hospital, Edegem, and Brussels University Hospital VUB, Belgium (Tassignon); Eye Clinic, Faculty of Medicine, University of Freiburg, Freiburg, Germany (Wacker); Department of Ophthalmology, University Medical Center Utrecht, Utrecht, the Netherlands (Dickman); Department of Cell Biology-Inspired Tissue Engineering, MERLN Institute for Technology-Inspired Regenerative Medicine, Maastricht, the Netherlands (Dickman).

## Abstract

**Purpose:**

To assess current prescription patterns in Descemet membrane endothelial keratoplasty (DMEK) in Europe.

**Setting:**

Countries affiliated with the European Cornea and Cell Transplantation Registry and the European Vision Institute Clinical Research Network.

**Design:**

Cross-sectional study (European survey).

**Methods:**

An electronic survey was distributed to 16 national societies to gather data on prescription patterns for DMEK. Responses were categorized by 3 clinical goals: prevention of postoperative endophthalmitis, graft rejection, and pupillary block and glaucoma.

**Results:**

Responses from 136 surgeons revealed that medication protocols mainly came from departmental protocols (54%) or personal experiences (48%) rather than national guidelines (22%) (multiple answers were allowed). Infection prevention primarily relied on intraoperative (72%) and postoperative (92%) antibiotics, with preoperative use less common (18%). Steroids were the mainstay for rejection prevention, used intraoperatively (59%), postoperatively (100%), and occasionally preoperatively (13%). Steroids were typically tapered to once daily after 6 months (46%) and discontinued after varying durations (65%). Dexamethasone was the preferred steroid. For high-risk DMEK, the most common approach was adding another topical (30%) or systemic immunosuppressive drug (24%), such as cyclosporine or mycophenolate. For graft rejection, most respondents increased topical steroid frequency (85%) or added (peri)bulbar steroid injections (42%). Pupillary block and glaucoma prophylaxis mainly involved intraoperative mydriatics (34%); intraocular pressure–lowering agents were rarely used (0.7% to 2.2%). For steroid-induced ocular hypertension, the common approach was switching to a lower-potency steroid (40%) or reducing steroid frequency (43%).

**Conclusions:**

Current prescription patterns in routine and high-risk DMEK vary significantly, reflecting the lack of standardized guidelines.

## Abstract 2

This comprehensive European survey reveals significant variability in DMEK prescription practices and highlights the need for standardized guidelines.

Descemet membrane endothelial keratoplasty (DMEK) is considered the gold standard for the surgical treatment of corneal endothelial diseases due to the fast and excellent recovery of vision and relatively low risk of rejection.^1–3^ However, preventing and treating graft rejection remains critical and requires balancing therapeutic effects with potential side effects because DMEK rejection signs are often subtle and challenging to recognize and monitor.^4^ The optimal protocol for rejection prevention and treatment is still unknown. Consequently, practitioners differ in their approaches to preventing and managing allograft rejection.^5^

Currently, there is limited evidence to guide treatment selection globally, and regimens are often based on previous transplantation techniques with a higher risk of rejection.^6–12^ Furthermore, treatment preferences may vary because of traditional protocols and medication availability.^13^ The literature on DMEK-specific prescription patterns is still scarce.^11,12^ This survey study evaluated the current prescription patterns of corneal surgeons in Europe specifically before, during, and after DMEK surgery, with a focus on preventing postoperative endophthalmitis, graft rejection, and pupillary block and glaucoma.

## METHODS

In March 2024, a survey of corneal surgeons practicing in Europe was conducted by electronic means. The steering group members of the European Cornea and Cell Transplantation Registry (ECCTR) and the European Vision Institute Clinical Research Network (EVICR.net) disseminated the electronic survey to all 16 distinct national societies affiliated with both ECCTR and EVICR.net, each responsible for promoting and supporting the collective interests and needs of corneal surgeons in their individual country. Participation was anonymous.

The survey was made up of 17 questions regarding respondents' clinical practices, including demographics, surgical volume, and prescription preferences and routes of administration before, during, and after DMEK. The final section of the survey focused on the management of high-risk DMEK, graft rejection, and steroid-induced ocular hypertension. Some questions allowed respondents to provide more information by selecting the “other” option (Table S1, available at http://links.lww.com/JRS/B393).

The collected responses were categorized and analyzed according to 3 primary clinical objectives: (1) prevention of postoperative endophthalmitis, (2) prevention of graft rejection, and (3) prevention of pupillary block and glaucoma. To assess infection prevention strategies, the use of prophylactic antibiotics before (Q5), during (Q8), and after surgery (Q10) was analyzed. Graft rejection prevention was evaluated based on steroid use across perioperative phases (Q5-6, Q8-11), tapering and discontinuation protocols (Q12-13), perspectives on high-risk cases (Q14-15), and preferred management of rejection signs (Q16). Pupillary block and glaucoma prevention was assessed by examining the use of mydriatics and prophylactic intraocular pressure (IOP)–lowering medications (Q5, Q8, Q10), as well as the stepwise management of steroid-induced ocular hypertension (Q17).

All survey questions were formatted using Qualtrics XM software (Qualtrics, LLC). Responses received up to 2 months after distribution of the survey were included in the analysis. Survey responses from countries with fewer than 5 respondents were excluded from the analysis, unless the country had fewer than 5 corneal transplant centers, to ensure a representative study sample across countries. Collected data were extracted from the Qualtrics platform and examined manually (ie, data reorganization and removal of double entries). Data were reported using descriptive statistics. The study was approved by the Medical Ethics Committee of the University Hospital Maastricht and Maastricht University (the Netherlands) (MREC ID 2023-3649).

## RESULTS

### Demographics and Clinical Routine Practice of Respondents

A total of 139 corneal surgeons (100%) completed the survey of whom 3 (2.2%) were excluded because they were the only respondents from countries with more than 4 corneal transplant centers (ie, Ireland, Russia, and Switzerland). Among the remaining 136 corneal surgeons (97.8%), the distribution across countries was as follows: Belgium (0.7%; n = 1), Denmark (0.7%; n = 1), France (13.2%; n = 18), Germany (19.1%; n = 26), Italy (9.6%; n = 13), the Netherlands (17.6%; n = 24), Portugal (9.6%; n = 13), Spain (13.2%; n = 18), and the United Kingdom (16.2%; n = 22).

**Figure 1.**
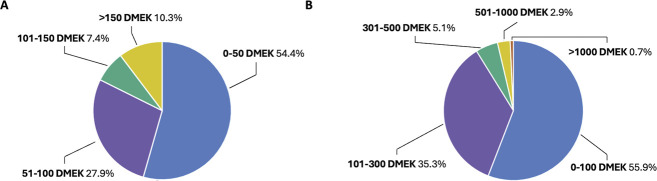
Distribution of annual routine DMEK procedures performed by surveyed corneal surgeons (*A*) and their affiliated institutes in 2023 (*B*). The percentage (%) of respondents is indicated next to each segment of the pie chart.

According to the survey, most respondents (54.4%; n = 74) conducted 50 or fewer routine DMEK procedures annually. The second largest group (27.9%; n = 38) reported performing 51 to 100 procedures, while the remaining surgeons (17.7%; n = 24) performed more than 100 procedures annually (Figure 1, A). Regarding the number of DMEK procedures performed in their affiliated institutes the previous year, most surgeons (55.9%; n = 76) reported 100 or fewer procedures. The second largest group (35.3%; n = 48) reported 101 to 300 procedures, while only a small proportion of surgeons (3.6%; n = 5) reported more than 500 procedures per year in their institution (Figure 1, B).

Regarding the clinical protocol for medication use in DMEK surgery, most surgeons reported following their personal protocol, derived from either departmental protocols (54.4%; n = 74) or their personal experience (47.8%; n = 65). Only a small proportion of surgeons based their protocol on national guidelines or preferred practice patterns (22.1%; n = 30) (multiple answers were allowed). Surgeons from different countries reported following national preferred practice patterns or guidelines: France (3.7%; n = 5), Germany (5.1%; n = 7), Italy (2.2%; n = 3), the Netherlands (4.4%; n = 6), Portugal (1.5%; n = 2), Spain (3.7%; n = 5), and the United Kingdom (1.5%; n = 2).

### Overview of Perioperative Medication Use

**Figure 2.**
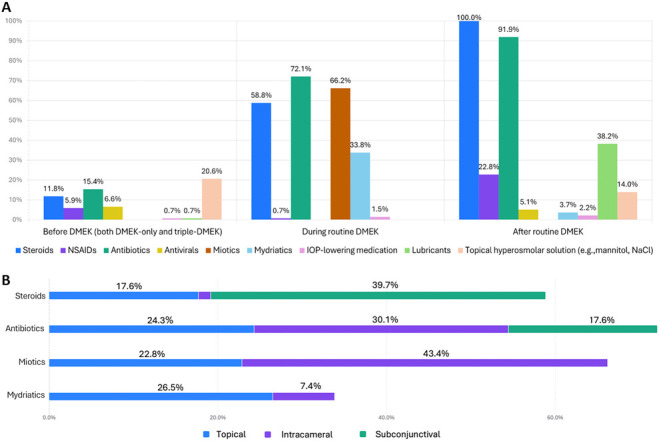
Distribution of medications prescribed before, during, and after DMEK procedures (*A*). Route of administration of medications during routine DMEK surgery (*B*). The percentage (%) of respondents is indicated above each bar.

An overview of medication use before, during, and after DMEK is presented in Figure 2, A. Most surgeons (59.6%; n = 81) did not prescribe any medication before DMEK. Among those who did, most (29.4%; n = 40) followed the same regimen for DMEK-only and triple-DMEK. In this group, the most prescribed medications were topical hyperosmolar solution (20.6%; n = 28), topical antibiotics (15.4%; n = 21), and topical steroids (11.8%; n = 16). A smaller group (11.0%; n = 15) applied different regimens. Among them, topical hyperosmolar solution (2.2%; n = 3), topical steroids (1.5%; n = 2), and topical miotics (0.7%; n = 1) were prescribed specifically in DMEK-only. Topical nonsteroidal anti-inflammatory drugs (NSAIDs) (5.9%; n = 8), topical antibiotics (2.2%; n = 3), and systemic antivirals (0.7%; n = 1) were limited to triple-DMEK.

During surgery, most surgeons used antibiotics (72.1%; n = 98), miotics (66.2%; n = 91), steroids (58.8%; n = 80), and mydriatics (33.8%; n = 47). The corresponding routes of administration are shown in Figure 2, B. DMEK was performed under general (36.0%; n = 49), retrobulbar (30.9%; n = 42), subtenon (28.7%; n = 39), and topical combined with intracameral anesthesia (3.7%; n = 5).

Postoperatively, all surgeons prescribed topical steroids (100%; n = 136). Antibiotics (91.9%; n = 125), lubricants (38.2%; n = 52), and NSAIDs (22.8%; n = 31) were also commonly used. Topical hyperosmolar solutions were less commonly prescribed (14.0%; n = 19), primarily in Germany (5.8%; n = 8).

### Protocols to Prevent Postoperative Endophthalmitis

Infection prevention was primarily addressed through intraoperative (72.1%; n = 98) and postoperative (91.9%; n = 125) antibiotic use (Figure 2, A). Preoperative topical antibiotics were prescribed by a minority of surgeons (17.6%; n = 24) of whom a small subset (2.2%; n = 3) limited their use to triple-DMEK cases. During surgery, antibiotics were predominantly administered intracamerally (30.1%; n = 41), followed by topical (24.3%; n = 33) and subconjunctival (17.6%; n = 24) administration (Figure 2, B).

### Protocols to Prevent Graft Rejection

Topical steroids were prescribed by all corneal surgeons (100%; n = 136) during the first month after DMEK surgery (Figure 2, A). Dexamethasone was the most frequently prescribed (87.5%; n = 119), followed by prednisolone (15.4%; n = 21) and fluorometholone (14.7%; n = 20) (multiple answers were allowed). Loteprednol was rarely prescribed (2.9%; n = 4), exclusively by surgeons from Germany (2.2%; n = 3) and Italy (0.7%; n = 1). Intraoperatively, steroids were administered by more than half of respondents (58.8%; n = 80), primarily through subconjunctival injection (39.7%; n = 54) (Figure 2, A). Intracameral preservative-free steroid administration was reported only in the United Kingdom (1.5%; n = 2) (Figure 2, B). Dexamethasone was most often used (70.9%; n = 56), followed by betamethasone (20.3%; n = 16), systemic (methyl)prednisolone (12.7%; n = 10), and triamcinolone (10.1%; n = 8). Preoperative topical steroid use was less common (13.2%; n = 18), with dexamethasone again being the most frequently prescribed (76.9%; n = 10), followed by prednisolone (23.1%; n = 3) and fluorometholone (7.7%; n = 1). Prednisolone was exclusively prescribed in Spain and fluorometholone in Portugal. Dexamethasone was the preferred perioperative steroid in all countries except the Netherlands, where betamethasone was the preferred intraoperative steroid (52.6%; n = 10).

**Figure 3.**
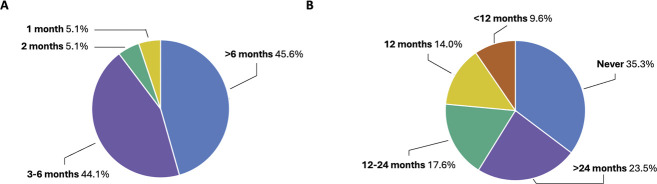
Distribution of steroid tapering regimen to once daily (*A*) and discontinuation of steroid prescription (*B*) after DMEK. The percentage (%) of respondents is indicated next to each segment of the pie chart.

Regarding tapering regimens, about half of surgeons tapered steroids down to once daily over more than 6 months (45.6%; n = 62) or within 3 to 6 months (44.1%; n = 60). Tapering within 1 or 2 months was infrequent (10.2%; n = 14) and observed across several countries (ie, France, Germany, Italy, the Netherlands, Portugal, Spain, and the United Kingdom) (Figure 3, A). Most surgeons (64.7%; n = 88) eventually stop prescribing steroids, while one-third (35.3%; n = 48) never discontinue steroid use. A small percentage of surgeons (9.6%; n = 13) in different countries discontinue steroid prescriptions within 12 months (ie, France, Italy, Portugal, Spain, and the United Kingdom) (Figure 3, B).

More than half of surgeons considered previous graft rejection (77.9%; n = 106), repeated corneal transplantation (67.6%; n = 92), and herpetic or other viral eye disease (64.7%; n = 88) as high-risk factors for graft rejection after DMEK. About half of surgeons viewed corneal vascularization as high-risk (48.5%; n = 66). By contrast, 16.2% (n = 22) of surgeons deemed DMEK intrinsically low risk for rejection, regardless of other factors. These perceptions were consistent across all surveyed countries. Other reported high-risk factors included complicated surgery with or without synechiae formation (1.5%; n = 2), an interrupted blood–aqueous barrier (0.7%; n = 1), bullous keratopathy (0.7%; n = 1), and noncompliance (0.7%; n = 1).

In cases of high-risk DMEK, the most common approach was adding another topical immunosuppressive drug (29.9%; n = 26) or a systemic immunosuppressive drug (24.1%; n = 21). HLA matching was less common (10.3%; n = 9), primarily practiced in the Netherlands (5.2%; n = 7), but also in France (0.7%; n = 1) and the United Kingdom (0.7%; n = 1). Among topical immunosuppressive agents, cyclosporine was the most frequently used (84.6%; n = 22), followed by tacrolimus (19.3%; n = 5) and prednisolone (3.8%; n = 1). Common systemic immunosuppressive agents included mycophenolate (44.4%; n = 8), prednisolone (38.9%; n = 7), unspecified steroids (11.1%; n = 2), tacrolimus (5.6%; n = 1), and methotrexate (5.6%; n = 1). Other strategies included increasing and prolonging the steroid tapering regimen (11.8%; n = 16), adding antiviral therapy (8.1%; n = 11), and starting steroids before surgery (0.7%; n = 1). Some surgeons (25.3%; n = 22) reported not modifying their practice in high-risk DMEK cases.

**Figure 4.**
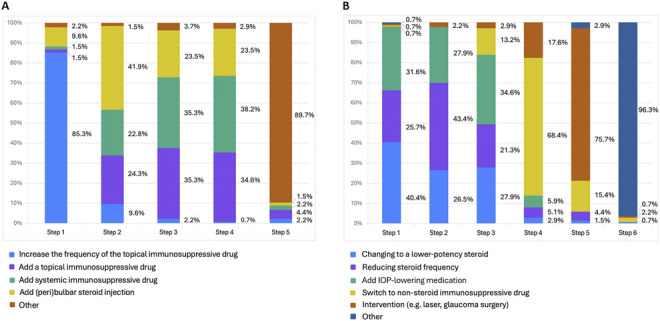
Distribution of the preferred step-by-step approach in case of graft rejection signs (*A*) and steroid-induced IOP elevation (*B*). The percentage (%) of respondents is indicated next to each bar.

When early signs of rejection occurred, increasing the frequency of topical immunosuppressive drugs was the most common initial treatment step (85.3%; n = 116), followed by (peri)bulbar steroid injection (41.9%; n = 57) as the second step. Adding either a topical or systemic immunosuppressive drug was both equally ranked as the third step (35.3%; n = 48), and adding a systemic immunosuppressive drug was the fourth (38.2%; n = 52) course of action (Figure 4, A). Other strategies included adding intracameral steroids (2.9%; n = 4), adding antiviral therapy (2.2%; n = 3), placing an intravitreal steroid implant (1.5%; n = 2), and obtaining an aqueous humor sample (0.7%; n = 1).

### Protocols to Prevent Pupillary Block and Glaucoma

Intraoperative mydriatic agents were used by one-third of surgeons (33.8%; n = 47), predominantly administered topically (26.5%; n = 36) (Figure 2, A and B). A small number of surgeons also reported using mydriatics postoperatively (3.7%; n = 5). Prophylactic prescription of IOP-lowering medication was rare before (0.7%; n = 1), during (1.5%; n = 2), and after surgery (2.2%; n = 3) (Figure 2, A). In cases of steroid-related ocular hypertension, changing to a lower-potency steroid was the most frequent step (40.4%; n = 55). Reducing the steroid frequency was the second step (43.4%; n = 59), followed by adding IOP-lowering medication as the third step (34.6%; n = 47). Switching to a non–steroid immunosuppressive drug was the fourth step (68.4%; n = 93), and intervention (eg, laser and glaucoma surgery) was the fifth step (75.7%; n = 103) (Figure 4, B).

## DISCUSSION

This survey-based study has revealed considerable variation in perioperative prescription patterns in DMEK among corneal surgeons across Europe. Certain practices were country-specific, while others were consistent across countries. Prescription patterns were based primarily on departmental protocols (54%) and personal experiences (48%). About 20% of surgeons reported adhering to preferred practice patterns or national guidelines. However, based on our knowledge and expert insights, not all surveyed countries, such as Portugal and Spain, have established such frameworks. These surgeons likely rely on literature, professional consensus, or guidelines from other countries.

Before DMEK, most surgeons (60%) did not prescribe any medication, unlike in cataract surgery, where topical steroids and NSAIDs are commonly used to reduce the risk of cystoid macular edema (CME).^14^ Given the prevalence of CME after DMEK, adopting similar regimens may be beneficial.^15^ However, the reported prevalence of CME comes from early DMEK studies and may not reflect current rates due to advances in the technique. In addition, animal studies suggest that preoperative steroids may improve corneal graft survival.^16^ Postoperatively, topical hyperosmolar eyedrops and ointments were mainly prescribed in Germany. However, a recent German randomized controlled trial (RCT) demonstrated that they had no clinically relevant effect on corneal edema and caused more discomfort than placebo.^17^

Antibiotic prophylaxis was widely adopted during (72%) and after surgery (91%). Preoperative use was more often reserved to triple-DMEK cases (2%), in line with common clinical practice for endophthalmitis prophylaxis in cataract surgery. The value of preoperative antibiotic prophylaxis, however, remains controversial.^18^

In DMEK, acute signs of graft rejection, such as Khodadoust lines, are rare, making detection subtle and challenging.^19^ Effective treatment is evidenced by improved corneal function and resolution of keratic precipitates.^4,19^ Topical steroids are either ketone-based (prednisolone, dexamethasone, and fluorometholone) or ester-based (loteprednol). The latter are locally hydrolyzed and less potent but result in a lower risk of ocular hypertension and (posterior subcapsular) cataract.^20^ Another potential side effect of steroid use is herpes virus reactivation.^21^

Dexamethasone was the preferred steroid before, during, and after DMEK surgery, although it raises the IOP in around 25% of patients postoperatively.^8^ Topical steroids vary in potency and corneal penetration both of which affect their effectiveness in preventing graft rejection.^22^ Lower-potency steroids such as fluorometholone and loteprednol are considered alternatives due to their reduced impact on IOP. However, fluorometholone's lower corneal penetration and loteprednol's rapid hydrolysis may limit their efficacy. Nonetheless, RCTs have shown that both steroids effectively prevent graft rejection with minimal impact on IOP.^23,24^ However, lower-potency steroids were rarely reported in our survey, possibly due to adherence to traditional practices based on older transplantation techniques with a higher risk of rejection or due to regional drug availability. Specifically, loteprednol was only prescribed in Germany and Italy, highlighting regional differences in steroid use and availability.

Steroids were typically tapered to once daily over more than 6 months postoperatively (46%) and continued for at least 1 year after DMEK (90%). The optimal dosing and tapering regimen remains unclear. Some recommend using topical steroid for 1 year after DMEK, while others suggest extending treatment for up to 2 years or even indefinitely.^6–8,10^ A prospective study reported a 6% rejection rate after stopping steroids at 1 year, with most cases being reversible. However, patient involvement in the decision to stop steroids, introduced biased, and treatment background during the first year was heterogeneous.^25^

There was no consensus on defining or managing high-risk DMEK. About 15% of surgeons considered DMEK to be intrinsically low risk for rejection, regardless of risk factors. However, limited research indicates higher rejection rates in DMEK for eyes with corneal neovascularization (4%) and repeat transplantation (7% to 28%), as well as higher graft failure rates in eyes with herpetic eye disease (18%).^21,26–28^ High-risk DMEK was managed with additional immunosuppressive therapy, either topical (30%) or systemic (24%). Topical agents were mainly cyclosporine (85%) and tacrolimus (19%), while systemic therapy commonly involved mycophenolate (44%). However, studies show no advantage in adding topical cyclosporine, whereas topical tacrolimus and systemic mycophenolate have been proven to be effective.^29,30^ Other valid options are systemic prednisolone (39%) and systemic tacrolimus (6%), although these were less often reported.^20^ HLA matching was mainly practiced in the Netherlands, with 1 report in the United Kingdom. However, this practice is no longer used in the United Kingdom according to expert opinion.

Graft rejection signs were primarily managed by increasing the frequency of topical immunosuppressive drugs (85%), supported by studies demonstrating effective control of DMEK graft rejection, while maintaining stable visual acuity and central corneal thickness and no further decrease in endothelial cell density.^8,25,31^ Less common but effective methods included preservative free intracameral steroids (3%) and, for severe cases, intravitreal steroid implants (1%) or intravenous pulse steroid therapy. However, our survey did not differentiate between types of systemic immunosuppressive therapies.^32–34^

IOP-lowering medication was rarely prescribed (0.7% to 2.2%) prophylactically. For steroid-induced ocular hypertension, the most common initial step was switching to a lower-potency steroid (40%). This approach seems appropriate, given the low rejection rate in DMEK, with RCTs confirming its safety and effectiveness in reducing steroid-induced ocular hypertension.^23,24^ When steroids are not an option, changing to a topical nonsteroid agent such as tacrolimus has been reported to be safe.^35^ For the prevention of pupillary block, 34% of surgeons use intraoperative mydriatics. However, we did not collect data on the relationship between the use of mydriatics and whether a peripheral iridotomy was performed.

To the authors' knowledge, this survey-based study is the first to focus on prescription patterns in DMEK. Survey responses were collected from a relatively large sample size (N = 136) across Europe. Nonetheless, the study has some limitations, including the unequal number of respondents from different member states. This was addressed by analyzing the results for each country separately and comparing them. In addition, only the primary country of practice was collected for reasons of privacy, and no other demographic data were demanded.

In summary, this European survey offers insights into different prescription preferences for infection prevention, graft rejection, and pupillary block and glaucoma in DMEK surgery. Despite the availability of lower-potency steroids with fewer side effects, dexamethasone remained the preferred steroid, and no precise tapering regimen was identified. These findings emphasize the need for standardized guidelines.

**WHAT WAS KNOWN**

- DMEK is the preferred surgical technique for corneal endothelial diseases.
- Preventing postoperative complications such as endophthalmitis, graft rejection, and pupillary block with glaucoma is essential for successful outcomes.
- Management of graft rejection requires balancing therapeutic effects with potential side effects because the signs of rejection can be subtle.

**WHAT THIS PAPER ADDS**

- To the authors' knowledge, this study is the first comprehensive survey to provide insights into perioperative medication practice in DMEK, focusing on the prevention of postoperative infection, graft rejection, and pupillary block and glaucoma.
- We found significant variation in practice patterns, indicating a need for standardized evidence-based guidelines.
